# Potential Efficacy of Metformin for Age-Related Macular Degeneration: A Systematic Review and Meta-Analysis

**DOI:** 10.1016/j.xops.2025.100741

**Published:** 2025-02-15

**Authors:** Matthew D. Huh, Simon N. Le, Kieran S. O'Brien, Jeremy D. Keenan, Jay M. Stewart

**Affiliations:** 1 Department of Ophthalmology, University of California, San Francisco, San Francisco, California; 2 Department of Ophthalmology, Zuckerberg San Francisco General Hospital and Trauma Center, San Francisco, California; 3 Francis I. Proctor Foundation for Research in Ophthalmology, University of California, San Francisco, San Francisco, California

**Keywords:** Metformin, Age-related macular degeneration, Systematic review, Meta-analysis

## Abstract

**Topic:**

Metformin, a widely used diabetes medication, has shown potential for treating age-related macular degeneration (AMD) due to its antioxidative, anti-inflammatory, and antiangiogenic properties. This study aims to systematically review and analyze the efficacy of metformin in reducing AMD prevalence.

**Clinical Relevance:**

Metformin's potential to serve as a treatment for AMD could significantly reduce the burden of vision loss, offering a cost-effective and widely accessible solution.

**Methods:**

A systematic search was conducted in OVID Embase, OVID MEDLINE, Cochrane Library, and Web of Science databases on May 2, 2024. Both observational and interventional studies were included if they involved oral metformin use before AMD diagnosis. Data were extracted and analyzed using a random-effects model meta-analysis, with subgroup analyses based on study design, AMD subtype, sex, and metformin dosage.

**Results:**

Eighteen observational studies were identified, which together included a total of 2 683 234 individuals. Nine studies had a case–control design, 7 were retrospective cohort studies, and 2 were cross-sectional studies. The meta-analysis revealed a significant reduction in the odds of AMD among metformin users (pooled odds ratio [OR] = 0.86, 95% confidence interval = 0.79–0.93, *P* = 0.0002, I^2^ = 90%). The association was significant in both patients with diabetes (pooled OR = 0.89) and without diabetes (pooled OR = 0.70), although only 2 studies reported nondiabetic ORs. Dose–response analysis revealed significant protective effects at low doses. Sensitivity analysis indicated that the removal of an outlier study did not alter the overall effect. Bias analysis using the Risk of Bias in Nonrandomized Studies of Interventions tool revealed significant risks of bias, particularly due to confounding.

**Conclusion:**

Although the current evidence suggests a potential protective role of metformin in AMD, all studies showing an effect of metformin have been observational and thus subject to bias. Randomized clinical trials are needed to determine the effectiveness of metformin for preventing the onset of AMD.

**Financial Disclosure(s):**

Proprietary or commercial disclosure may be found in the Footnotes and Disclosures at the end of this article.

Age-related macular degeneration (AMD) is a leading cause of blindness, especially in individuals over the age of 65 years. This condition is characterized by the progressive degeneration of the macula affecting central vision, affecting roughly 20 million people in the United States and 200 million globally.^1^ Age-related macular degeneration is classified into 2 forms: non-neovascular (also known as nonexudative) and neovascular (also known as exudative).^2^ Non-neovascular AMD is more prevalent and is defined by the accumulation of drusen, extracellular deposits found beneath retinal layers.^3^ This form of AMD can progress slowly and can be less impactful on vision, except in its later stages. In contrast, neovascular AMD is characterized by abnormal blood vessel growth under the retina, which can lead to more severe vision loss.

### Problem Overview

Treatment options for AMD remain limited. For non-neovascular AMD, the Age-related Eye Disease Study 2 formulation of vitamins has shown efficacy in slowing disease progression, though no treatments currently exist to reverse the condition.^4^ As for neovascular AMD, although treatments such as anti-VEGF agents have demonstrated effectiveness, they pose limitations due to the invasive and costly nature of therapy options. Given the limitations of current therapies, prevention represents a pivotal strategy for reducing AMD prevalence and enhancing patient outcomes.^5^ Metformin, widely used in the management of diabetes mellitus (DM),^6^ has demonstrated antioxidative,^7^ anti-inflammatory,^8^ and antiangiogenic effects,^9^ suggesting potential relevance to AMD-related pathways. Preliminary studies suggest that metformin could influence cellular mechanisms associated with AMD progression, offering a promising avenue for therapeutic exploration to potentially slow or prevent the onset of this vision-impairing condition.

Previous meta-analyses found a protective effect of metformin on AMD.^10^^,^^11^ Since that time, additional literature on the topic has been published, providing a richer dataset that allows more subgroup analyses and a larger sample size. For these reasons, it is important to conduct an updated systematic review and meta-analysis to elucidate the impact of metformin use on AMD, drawing upon all currently accessible evidence.

## Methods

This study adhered to the Preferred Reporting Items for Systematic Reviews and Meta-Analyses 2020 guidelines.^12^ The protocol for this review was registered on the International Prospective Register of Systematic Reviews under the ID CRD42024541360. Due to the nature of the study, institutional review board approval was waived. All research adhered to the tenets of the Declaration of Helsinki.

### Study Selection

In this meta-analysis, we included both observational studies (such as case–control, cohort, and cross-sectional studies) and interventional studies that measured the association between metformin and incident AMD. We excluded studies that only assessed the association of metformin with the progression of AMD, were not in English, were not conducted in humans, did not consist of full articles providing sufficient data, or did not provide appropriate outcome data. Although most participants had some form of diabetes due to the retrospective nature of most observational studies and the fact that metformin is primarily a diabetes medication, diabetes was not a requirement for inclusion. The intervention of interest was oral metformin, with the experimental group consisting of metformin users compared to nonusers. The primary outcome measures analyzed were odds ratios (ORs) and hazard ratios (HRs) representing associations between oral metformin and AMD.

### Search Strategy

Our search strategy involved systematically retrieving papers from multiple databases, including OVID Embase, OVID MEDLINE, Cochrane Library, and Web of Science. The search was conducted on May 2, 2024, using specific search criteria outlined in Table S1 (available at www.ophthalmologyscience.org).

### Data Extraction

Two reviewers (authors M.D.H. and S.N.L.) independently conducted the initial screening of titles and abstracts identified in the preliminary search to determine potential relevance. Subsequently, full texts of these potentially eligible studies were reviewed to confirm their inclusion. Discrepancies in the selection process were addressed through discussion between the 2 reviewers, with a third reviewer (author J.M.S.) consulted if consensus could not be achieved. Data extraction from the included studies was performed independently by M.D.H. and S.N.L., collecting information on authorship, study setting, study type, participant numbers, AMD diagnosis, participant characteristics, and covariates. Any discrepancies in data extraction were resolved by discussion, with J.M.S. serving as an arbitrator if necessary. The risk of bias in individual studies was independently assessed by 2 reviewers (authors J.D.K. and K.S.O.) using the Risk of Bias in Nonrandomized Studies of Interventions (ROBINS-I) assessment tool.^13^ Differences in bias assessments were resolved through consensus between these 2 reviewers.

### Data Synthesis and Statistical Analysis

We conducted meta-analyses using The Cochrane Collaboration Review Manager (RevMan) software, version 5.4.1. Age-related macular degeneration was reported as a dichotomous outcome, with pooled estimates expressed as ORs and HRs with 95% confidence intervals (CIs). Statistical significance was defined as a *P* value <0.05. The ORs used in the meta-analyses were derived from covariate-adjusted, multivariable analyses associated with the sole use of metformin. These ORs were pooled using a generic inverse variance statistical approach with the DerSimonian–Laird random-effects model. A random-effects model was chosen due to the significant heterogeneity observed, its ability to accommodate different study designs, and our objective to generalize the findings.^14^

Between-study heterogeneity was assessed using the I^2^ statistic, which quantifies the proportion of total variation across studies attributable to heterogeneity rather than chance. I^2^ values exceeding 75% were considered indicative of high heterogeneity.^15^ Subgroup analyses were performed to explore and potentially mitigate heterogeneity by examining whether specific study characteristics could explain the observed variability in effect sizes.

A sensitivity analysis was performed to assess the robustness of the meta-analysis findings. This was done using a funnel plot to identify studies at risk of publication bias, which occurs when studies with significant or positive results are more likely to be published. Based on the asymmetry of the plot, studies identified as having a relatively high risk of bias were excluded from subsequent analysis.^16^

## Results

### Study Selection

**Figure 1:**
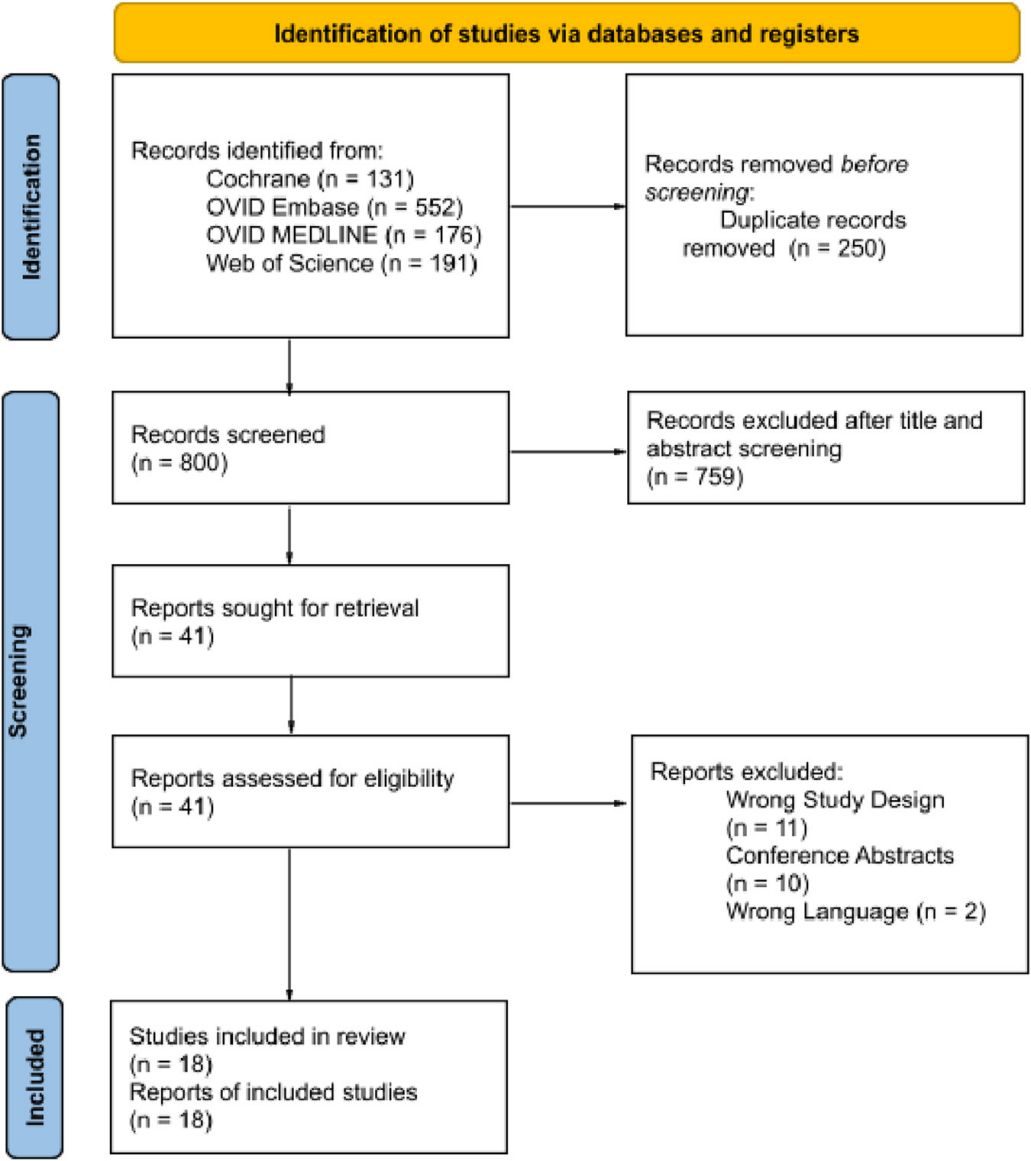
Preferred Reporting Items for Systematic Reviews and Meta-Analyses flowchart summarizing the study selection process. This flowchart depicts the systematic identification and screening of studies included in the meta-analysis. The initial database search yielded 800 records, of which 41 full-text articles were assessed for eligibility, and 18 studies were included in the final analysis. Each step of the selection process, including the reasons for exclusion at each stage, is outlined.

The Preferred Reporting Items for Systematic Reviews and Meta-Analyses flowchart (Fig 1) summarizes the results of our data extraction. After the exclusion of duplicate records, an abstract and title screening was performed for 800 studies. Forty-one texts were retrieved and assessed for eligibility, and 18 studies meeting the eligibility criteria were included in the quantitative meta-analysis.^17^, ^18^, ^19^, ^20^, ^21^, ^22^, ^23^, ^24^, ^25^, ^26^, ^27^, ^28^, ^29^, ^30^, ^31^, ^32^, ^33^, ^34^

### Characteristics of Included Studies

Study characteristics and relevant outcomes are presented in Table 1. A total of 2 683 234 individuals were reported in these studies, although 5 studies used the same patient database.^17^^,^^23^^,^^24^^,^^26^^,^^30^ Of the 18 studies, 9 had a case–control design,^17^, ^18^, ^19^^,^^21^^,^^23^, ^24^, ^25^, ^26^^,^^30^ 7 were retrospective cohort studies,^22^^,^^27^^,^^29^^,^^31^, ^32^, ^33^, ^34^ and 2 were cross-sectional studies.^20^^,^^28^ Fourteen studies measured ORs,^17^, ^18^, ^19^, ^20^, ^21^, ^22^, ^23^, ^24^, ^25^, ^26^, ^27^, ^28^, ^29^, ^30^ and 4 studies measured HRs.^31^, ^32^, ^33^, ^34^ Eleven studies were conducted in the United States,^17^, ^18^, ^19^, ^20^^,^^23^^,^^24^^,^^26^, ^27^, ^28^^,^^30^^,^^31^ 5 studies were conducted in Asia,^21^^,^^22^^,^^25^^,^^32^^,^^34^ and 2 studies were conducted in Europe.^29^^,^^33^ Fifteen studies classified AMD according to *International Classification of Diseases* codes,^17^, ^18^, ^19^^,^^21^, ^22^, ^23^, ^24^, ^25^, ^26^, ^27^, ^28^^,^^30^, ^31^, ^32^^,^^34^ and 3 did so with medical records.^20^^,^^29^^,^^33^ Eleven studies recruited patients with either type 1 or type 2 DM,^18^, ^19^, ^20^^,^^23^, ^24^, ^25^, ^26^, ^27^, ^28^^,^^30^^,^^31^ 6 studies recruited patients with only type 2 DM,^21^^,^^22^^,^^29^^,^^32^, ^33^, ^34^ and one study recruited only patients without DM.^17^ Sixteen studies examined the outcome of any subtype of AMD,^17^, ^18^, ^19^, ^20^, ^21^, ^22^^,^^25^, ^26^, ^27^, ^28^, ^29^, ^30^, ^31^, ^32^, ^33^, ^34^ whereas the remaining 2 studies examined only non-neovascular and neovascular AMD, respectively.^23^^,^^24^ Covariate data are detailed in Table S2 (available at www.ophthalmologyscience.org).

**Table 1:** Study Characteristics and Relevant Outcomes

Studies	Country	Study Period	Databases	Age Mean (SD), yrs	No of Participants (Exposed/Nonexposed)	Follow-Up Periods, yrs	Definition of Metformin Use	Definition of AMD Diagnosis	OR/HR Value
Aggarwal et al (2024)	United States	2008–2017	Merative MarketScan and Medicare	75.1 (10.4)	53 554/458 666	N/A	N/A	ICD-9 and ICD-10 codes	OR: 0.83 (0.78–0.88)
Blitzer et al (2021)	United States	2008–2017	Merative MarketScan and Medicare	74.9 (10.3)	80 811/543 969	N/A	National Drug Code	ICD-9 and ICD-10 codes	OR: 0.95 (0.93–0.97)
Brown et al (2019)	United States	2011–2017	University of Florida Integrated Data Repository	75.5 (9.0)	695/7903	N/A	RxNorm IDs	ICD-9 codes	OR: 0.70 (0.49–0.98)
Chen et al (2019)	Taiwan	2001–2013	Taiwan Longitudinal Health Insurance Database	56.1 (12.6)	45 524/22 681	6.3 (3.7)	National Drug Code	ICD-9 codes	HR: 0.54 (0.50–0.58)
Domalpally et al (2003)	United States	1996–2018	Diabetes Prevention Program Outcomes Study	68.8 (9.0)	549/1043	16 (1)	N/A	SD-OCT image analysis	OR: 0.97 (0.84–1.12)
Eton et al (2002)	United States	2016–2021	Clinformatics Data Mart	67.5 (8.9)	116 114/841 111	N/A	1000 mg/day	ICD-10 codes	HR: 1.08 (1.04–1.12)
Gokhale et al (2022)	United States	1995–2019	IMRD-UK	62.8 (11.6)	154 016/19 673	5.7 (4.1)	Medical records	Database Read codes	HR: 1.02 (0.92–1.12)
Huang et al (2023)	Taiwan	2002–2013	Longitudinal Health Insurance Database	62 (8.8)	377 873/350 825	1 (5)	Anatomical Therapeutic Chemical Code	ICD-9 and ICD-10 codes	OR: 1.004 (0.98–1.02)
Jiang et al (2022)	China	2015–2020	China–Japan Friendship Hospital EMR	67 (No SD reported)	209/115	N/A	Daily average does >250 mg	ICD-10 codes	OR: 0.23 (0.13–0.38)
Kaufmann et al (2023)	United States	2008–2017	Merative MarketScan and Medicare	74.2 (5.03)	52 008/336 117	N/A	N/A	ICD-9 and ICD-10 codes	OR: 0.97 (0.95–1.00)
Khanna et al (2024)	United States	2003–2019	Merative MarketScan and Medicare	78.4 (9.7)	20 636/153 212	N/A	National Drug Code	ICD-9 and ICD-10 codes	OR: 0.95 (0.91–0.98)
Moir et al (2024)	United States	2016–2021	Merative MarketScan and Medicare	82.2 (9.6)	1149/1277	N/A	N/A	ICD codes	OR: 0.88 (0.79–0.99)
Lee et al (2019)	Korea	2012–2015	NHIS-NEC	66.4 (5)	10 768/14 840	N/A	Prescription history	ICD-10 codes	OR: 1.15 (0.91–1.45)
Shaw et al (2024)	United States	2006–2017	Merative MarketScan and Medicare	74.8 (9.6)	76 968/83 791	N/A	National Drug Code	ICD-9 and ICD-10 codes	OR: 0.94 (0.93–0.96)
Starr et al (2022)	United States	2004–2013	Rochester Epidemiology Projects Records	76.5 (10)	160/1352	4.55 (2.9–7.2)	N/A	ICD-9 codes	OR: 0.60 (0.4–0.91)
Stewart et al (2020)	United States	2012–2019	UCSF-EMR	71.5 (7.5)	1837/1135	N/A	Medical Record	ICD-9 and ICD-10 codes	OR: 0.7 (0.55–0.88)
Tseng (2023)	Taiwan	1999–2005	Taiwan NHI	65 (0.3)	13 303/13 303	N/A	N/A	ICD-9 codes	HR: 0.756 (0.673–0.85)
Vergroesen et al (2022)	The Netherlands	1990–2014	Rotterdam Study	65.2 (9.8)	617/10 376	8.2 (1.1–21.7)	Anatomical Therapeutic Chemical Code	Rotterdam Classification	OR: 0.69 (0.48–0.98)

AMD = age-related macular degeneration; HR = hazard ratio; ICD = *International Classification of Diseases*; IMRD-UK: IQVIA Medical Research Data - United Kingdom; N/A = not applicable; NHI = National Health Insurance; NHIS-NEC = National Health Insurance Service - National Health Examination Cohort; OR = odds ratio; SD = standard deviation; UCSF-EMR = University of California, San Francisco - Electronic Medical Record.

### Bias Analysis

**Figure 2:**
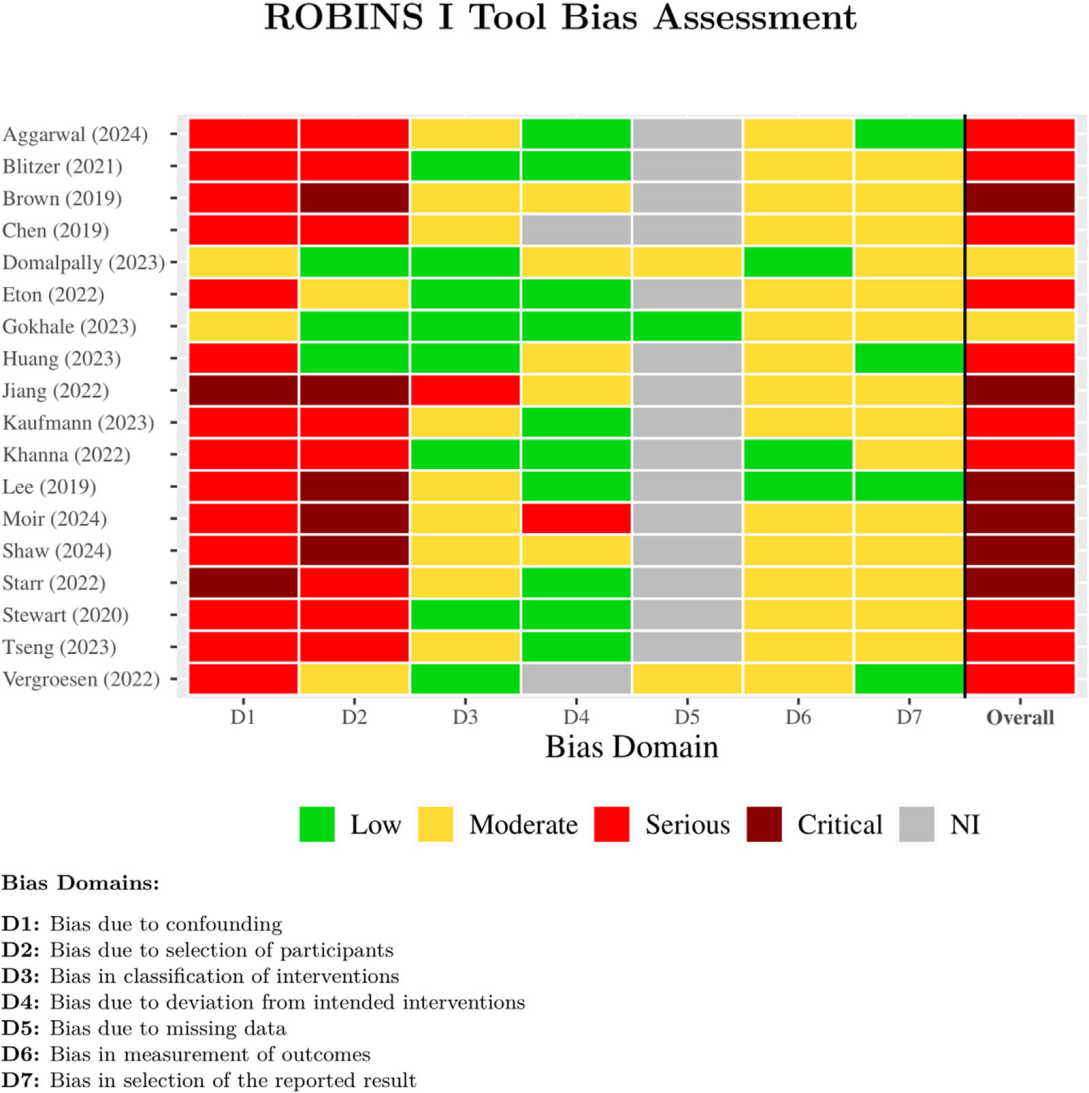
Risk of bias assessment using the ROBINS-I tool. The bar chart shows the proportion of included studies rated as low, moderate, serious, or critical risk of bias across 7 domains, including confounding, selection of participants, and outcome measurement. Most studies exhibited a serious risk of bias in the confounding domain due to the observational design. The findings underscore the need for cautious interpretation and highlight key areas for methodological improvement in future research. ROBINS-I = Risk of Bias in Nonrandomized Studies of Interventions.

Bias was assessed using the ROBINS-I tool, developed by Cochrane.^13^ The ROBINS-I tool is a comprehensive method for evaluating the risk of bias in nonrandomized studies, addressing various bias domains, including bias due to confounding (D1), bias due to selection of participants (D2), bias in classification of interventions (D3), bias due to deviations from intended interventions (D4), bias due to missing data (D5), bias in measurement of outcomes (D6), and bias in the selection of the reported result (D7). We selected the ROBINS-I tool over the Risk of Bias in Non-randomized Studies of Exposures tool because the former is specifically designed for nonrandomized studies of interventions, making it more suitable for our dataset, which includes observational studies with intervention components. All studies had considerable bias across multiple domains, underscoring the importance of careful interpretation of the results (Fig 2).

### Association of Oral Metformin Use with AMD

**Figure 3:**
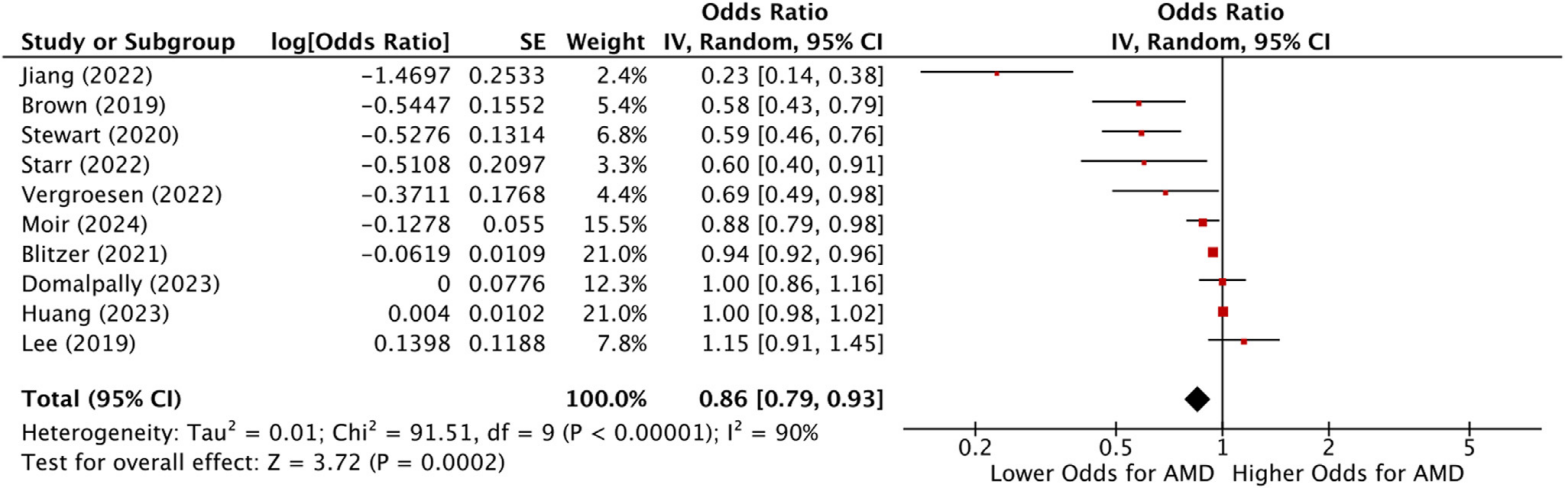
Forest plot of pooled ORs for the association between metformin use and AMD. The plot shows individual study ORs and their 95% CIs, along with the overall pooled estimate (OR = 0.86, 95% CI = 0.79–0.93, *P* = 0.0002, I^2^ = 90%). The diamond at the bottom represents the pooled estimate, indicating a statistically significant protective effect of metformin use against AMD. The high I^2^ statistic reflects considerable heterogeneity among studies. AMD = age-related macular degeneration; CIs = confidence intervals; ORs = odd ratios.

**Figure 4:**
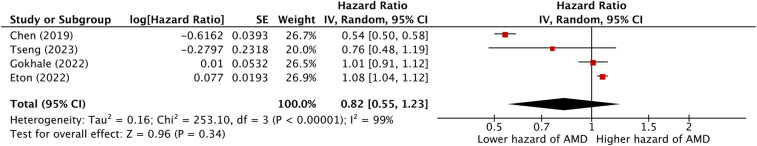
Forest plot of pooled HRs for the progression of AMD among metformin users. Individual study HRs and their 95% CIs are displayed alongside the pooled estimate (HR = 0.82, 95% CI = 0.55–1.23, *P* = 0.34, I^2^ = 99%). The wide confidence intervals and high I^2^ value suggest variability and limited consensus across studies. The results indicate no statistically significant association between metformin use and AMD progression. AMD = age-related macular degeneration; CIs = confidence intervals; HRs = hazard ratios.

A total of 18 studies consisting of 2 683 234 individuals reported multivariable-adjusted ORs and HRs for oral metformin (1 407 508 and 1 275 726 patients, respectively), although 6 of these studies^17^^,^^18^^,^^23^^,^^24^^,^^26^^,^^30^ used the same underlying Merative Marketscan and Medicare database. To avoid the problem of duplicate publication, only 2 of these papers with the largest nonoverlapping study populations were included in the main analysis.^18^^,^^30^ Meta-analysis showed a significant decrease in the odds of AMD resulting from oral metformin usage. Odds ratios and HRs were pooled separately, giving a pooled OR of 0.86 (95% CI = 0.79 to 0.93, *P* = 0.0002, I^2^ = 90%; Fig 3) and a pooled HR of 0.82 (95% CI = 0.55 to 1.23, *P* = 0.34, I^2^ = 99%; Fig 4).

Among the study types, case–control studies had a pooled OR = 0.93, 95% CI = 0.90 to 0.97, *P* < 0.00001, I^2^ = 86%, while cross-sectional (*P* = 0.27) and cohort studies (*P* = 0.26) had nonsignificant pooled OR and CIs (Fig S1, available at www.ophthalmologyscience.org). In studies measuring ORs specific to AMD subtype, the pooled OR for non-neovascular AMD was 0.86 (95% CI = 0.75 to 0.97, *P* = 0.02, I^2^ = 86%), and the pooled OR for neovascular AMD was 0.95 (95% CI = 0.91 to 1.00, *P* = 0.06, I^2^ = 0%; Fig S2, available at www.ophthalmologyscience.org). Significant associations were observed in patients with diabetes (pooled OR = 0.89, 95% CI = 0.84–0.95, *P* = 0.0003, I^2^ = 98%) but not in patients without diabetes (pooled OR = 0.70, 95% CI = 0.45–1.08, *P* = 0.11, I^2^ = 74%). Notably, there were only 2 studies that measured ORs in only patients with diabetes,^17^^,^^30^ and one of these^30^ only included patients with geographic atrophy (GA) (Fig S3, available at www.ophthalmologyscience.org).

Five studies^17^^,^^18^^,^^21^^,^^23^^,^^24^ tracked ORs in different metformin dosage categories, measured in cumulative grams of metformin per 2 years. Because Aggarwal et al, Blitzer et al, Kaufmann et al, and Khanna et al take data from the same database, only Blitzer et al and Huang et al were included in this analysis. Blitzer et al defined low dosage as 1 to 270 g/2 years, medium dosage as 271 to 600 g/2 years, high dosage as 601 to 1080 g/2 years, and very high dosage as >1080 g/2 years. Huang et al defined low dosage as 0 to 240 g/2 years, medium dosage as 240 to 720 g/2 years, high dosage as 720 to 1200 g/2 years, and very high dosage as >1200 g/2 years. A significant association was found for the low-dose group (pooled OR = 0.92, 95% CI = 0.89–0.94, *P* < 0.00001, I^2^ = 0%). Other dosage categories did not show any significant association (medium dose: pooled OR = 0.94, 95% CI = 0.85 to 1.04, *P* = 0.21, I^2^ = 72%; high dose: pooled OR = 0.96, 95% CI = 0.92 to 1.00, *P* = 0.06, I^2^ = 18%; very high dose: pooled OR = 1.16, 95% CI = 0.86 to 1.56, *P* = 0.33, I^2^ = 82% (Fig S4, available at www.ophthalmologyscience.org).

### Sensitivity Analysis

**Figure 5:**
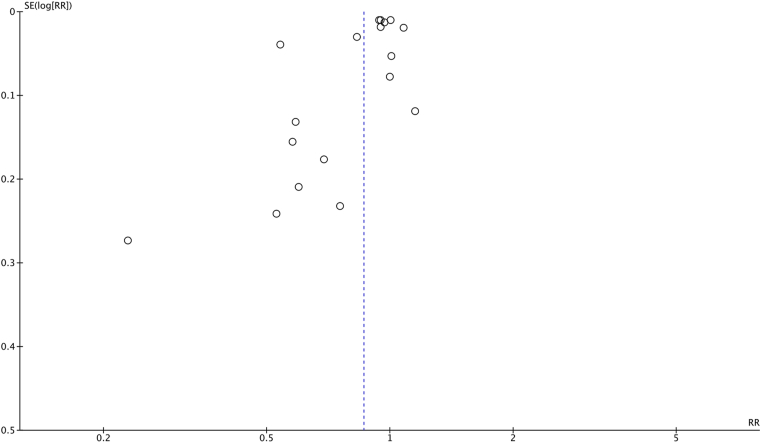
Funnel plot for sensitivity analysis assessing publication bias. The plot depicts the distribution of effect sizes (ORs) vs. their standard errors. Asymmetry is observed, with one study (Jiang et al) appearing to be an outlier. This study's exclusion in the sensitivity analysis resulted in a marginally higher pooled OR (0.90, 95% CI = 0.84–0.96, *P* = 0.003) and a reduction in heterogeneity (I^2^ = 87%), suggesting that publication bias may partially influence the findings. CIs = confidence intervals; ORs = odd ratios.

The funnel plot (Fig 5) showed asymmetry, suggesting potential publication bias, particularly highlighted by the study by Jiang et al, represented by the point furthest to the left. This indicates that this study may be an outlier, contributing disproportionately to the observed heterogeneity. A sensitivity analysis that omitted this study had a similar result, with an OR of 0.90 (95% CI: 0.84–0.96, *P* = 0.003) and an I^2^ value 3% lower than the original analysis (Fig S5, available at www.ophthalmologyscience.org).

## Discussion

Our meta-analysis found a significant association between oral metformin use and reduced odds of AMD (pooled OR = 0.86, 95% CI = 0.79–0.93, *P* = 0.0002, I^2^ = 90%). However, high heterogeneity (I^2^ = 90%) underscores the need for cautious interpretation and further research to validate these findings.

Metformin could have a therapeutic effect against AMD through various mechanisms. Metformin can reduce drusen formation by activating adenosine monophosphate-activated protein kinase, which in turn activates liver X receptor, leading to increased expression of adenosine triphosphate-binding cassette transporters adenosine triphosphate-binding cassette A1 and adenosine triphosphate-binding cassette G1.^35^ Metformin's anti-inflammatory effects lie in its ability to impede the differentiation of monocytes into macrophages, a process that significantly hastens atherosclerosis by promoting inflammatory conditions within the vessel.^36^ This drug can hinder tumor necrosis factor–induced expression of intercellular adhesion molecule 1 and monocyte chemoattractant protein 1 while also inhibiting the proliferation, migration, and tube formation of endothelial cells, thereby preventing retinal neovascularization.^37^ Metformin's antioxidative effects come from its ability to downregulate the production of reactive oxygen species by nicotinamide adenine dinucleotide ubiquitin oxidoreductase.^38^ It is these processes that make metformin a strong candidate to be implemented as a therapeutic treatment for AMD.

Given that metformin is primarily prescribed for diabetes, most studies in this meta-analysis included patients with diabetes, complicating the isolation of metformin's effects on AMD from the inherent risks posed by diabetes itself. Diabetes is a known risk factor for AMD due to its impact on vascular health.^39^ Consequently, patients with diabetes who use metformin might show an apparent protective effect that is difficult to separate from their underlying condition. The observational design of all reviewed studies further limits our ability to control for this confounding factor. Notably, only one study focused solely on patients without diabetes,^17^ and only one other study^30^ conducted a subgroup analysis on patients without diabetes. This subgroup analysis reported a greater association with metformin, with an OR of 0.58 (95% CI = 0.33 to 0.83).

Our findings, based on ORs reported in 5 included studies,^17^^,^^18^^,^^21^^,^^23^^,^^24^ indicate that significant associations were present in the low-dose category but not in the medium-, high-, or very-high-dose category, suggesting a possible optimal therapeutic range. Although this may suggest that lower doses cause protective effects, it may also be due to patients requiring low doses being healthier and thus being less likely to develop AMD. This dose–response relationship highlights the need for future randomized trials to prioritize identifying the optimal dosage for AMD treatment. Further mechanistic studies are warranted to elucidate the pathways through which metformin exerts its effects at different dosages, thereby informing better clinical practices and optimizing patient care.

To date, only one randomized clinical trial has investigated the effect of metformin on AMD.^40^ This study did not meet the eligibility criteria of the present meta-analysis because it assessed for progression among participants with existing GA. Nonetheless, its findings remain noteworthy. The trial randomized 66 patients aged ≥55 years without diabetes, all of whom had GA from atrophic AMD in one or both eyes. Of the participants, 34 (57 eyes) were assigned to the observation group, while 32 (53 eyes) received oral metformin. The primary endpoint was the annualized rate of GA progression, which did not show a significant association with metformin treatment. However, the small sample size may have limited the statistical power to detect a treatment effect. Although GA progression occurs in the later stages of AMD,^41^ metformin may still play a role in mitigating other factors such as the onset and progression of hypertransmission defects (increased signal penetration on optical coherence tomography scanning in areas of incipient atrophy), drusen size, or neovascularization earlier in the disease course. These areas warrant further investigation to fully understand metformin's potential in AMD management.

### Weaknesses

This study has several limitations that should be considered. This meta-analysis included only retrospective observational studies. The observational nature of the included studies introduced bias, particularly related to confounding factors like the presence and severity of diabetes, which complicated the assessment of the true association between metformin and AMD. A misclassification error may have affected the observed measures of association. The lack of a standardized protocol in these studies resulted in a heterogeneous metformin intervention that may have biased the measures of association toward the null.

While the current observational evidence suggests a potential protective role of metformin in AMD, randomized trials are required to confirm these findings and determine the clinical applicability and appropriate dosing of metformin as a preventive treatment for AMD. Additionally, acknowledging that the current body of research predominantly includes patients with diabetes, it is essential to emphasize that this specificity limits the generalizability of the findings to nondiabetic populations. Future investigations should therefore aim to explore the effects of metformin on AMD in a broader demographic to validate its efficacy and applicability across diverse patient groups. If found to be effective in randomized trials, metformin could play a substantial role in AMD management, especially given its low cost, wide availability, and favorable safety profile. The need for trials is all the more urgent, given the paucity of alternative treatment options for patients with AMD.
