# Supplementary figures and images for: Potential Efficacy of Metformin for Age-Related Macular Degeneration: A Systematic Review and Meta-Analysis
Source: Ophthalmol Sci. 2025 Feb 15;5(4):100741. doi: 10.1016/j.xops.2025.100741 (PMC11994399; doi:10.1016/j.xops.2025.100741)

Supplementary Figure 1: Study Type Forest Plot

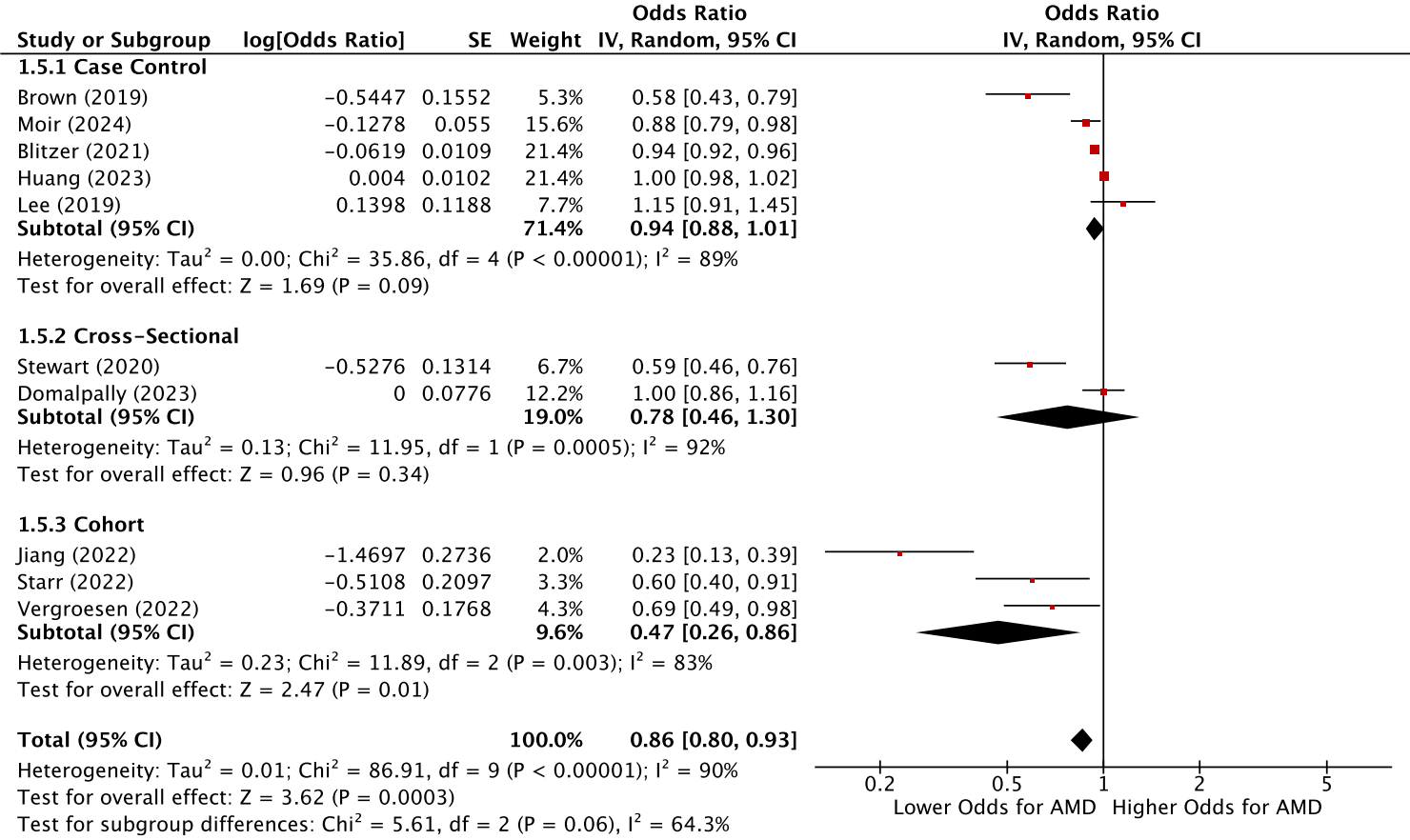

Supplement: Figure S1 [file mmc1.pdf]

Supplementary Figure 2: AMD Type Forest Plot

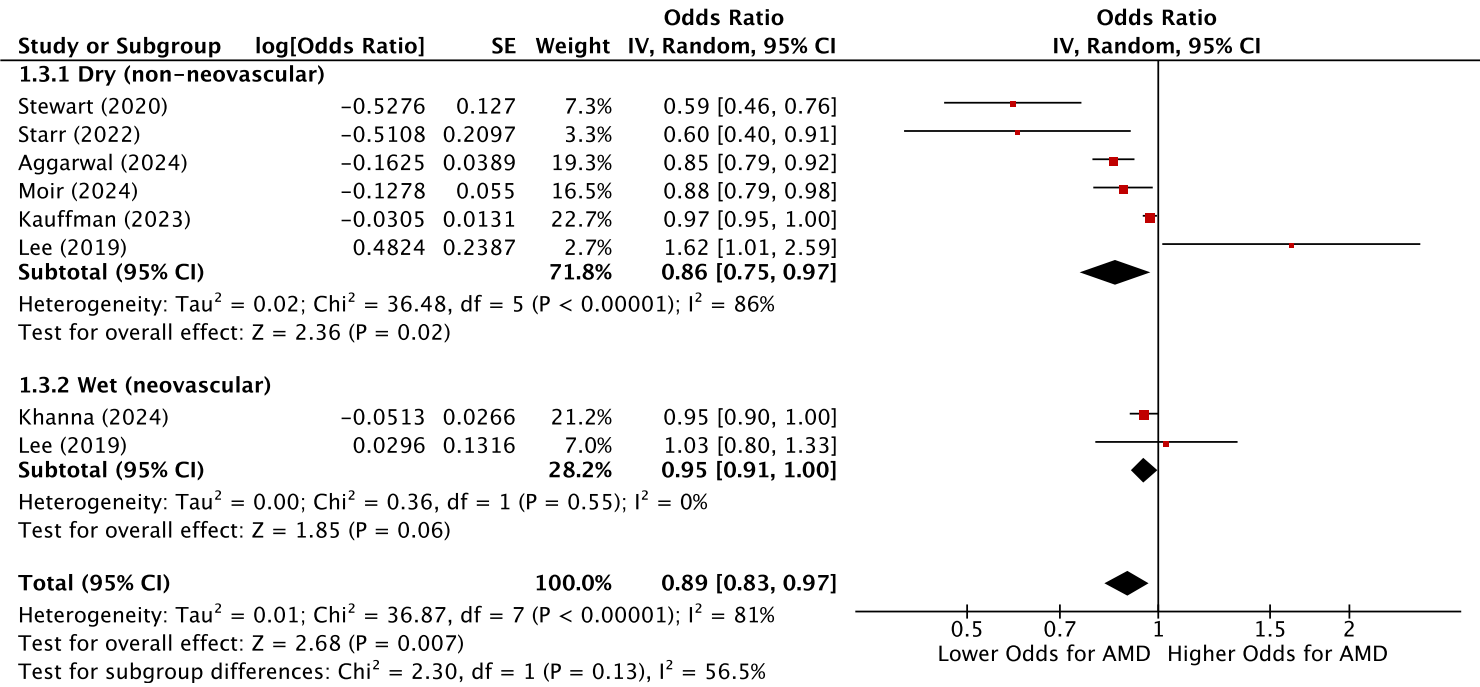

Supplement: Figure S2 [file mmc2.pdf]

Supplementary Figure 3: Diabetes Forest Plot

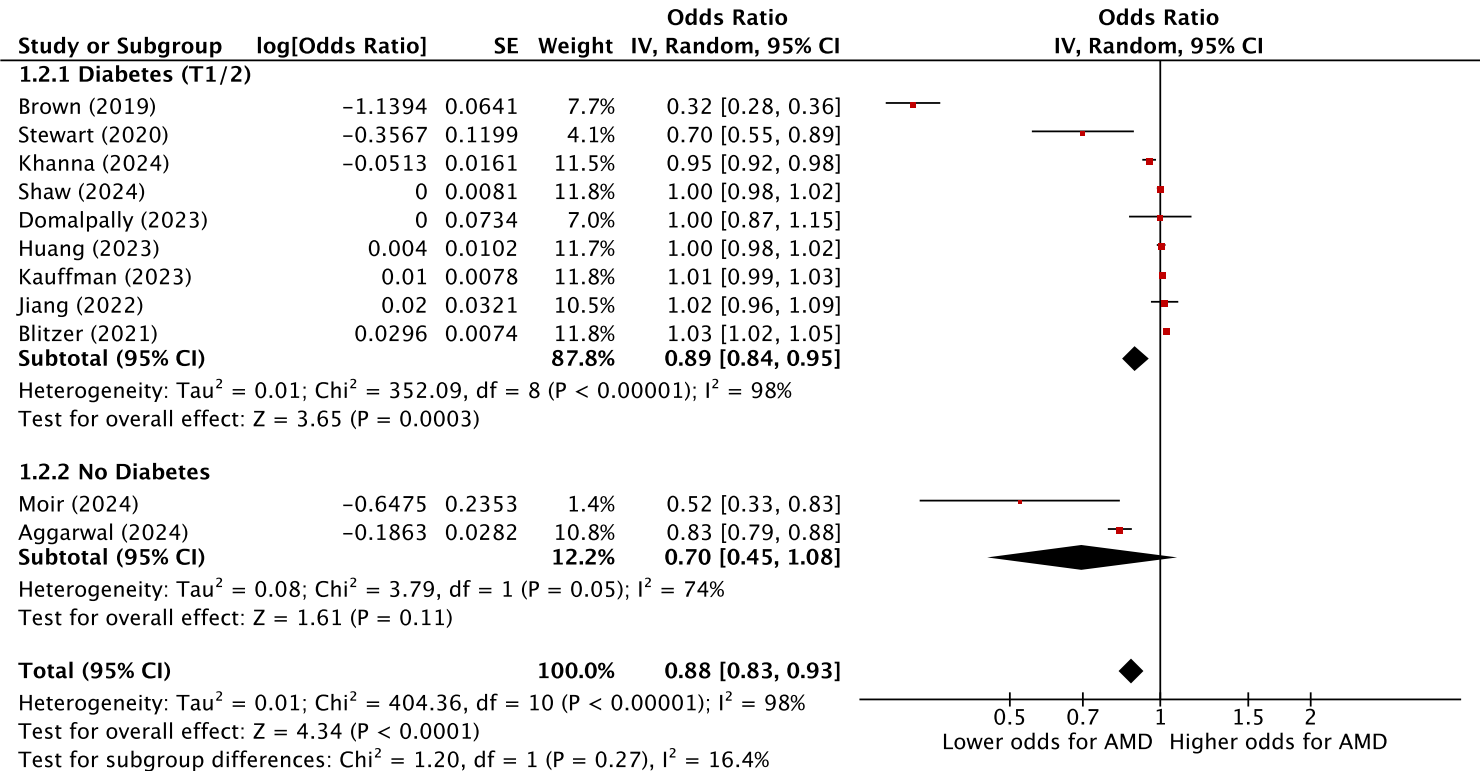

Supplement: Figure S3 [file mmc3.pdf]

Supplementary Figure 4: Dosage Forest Plot

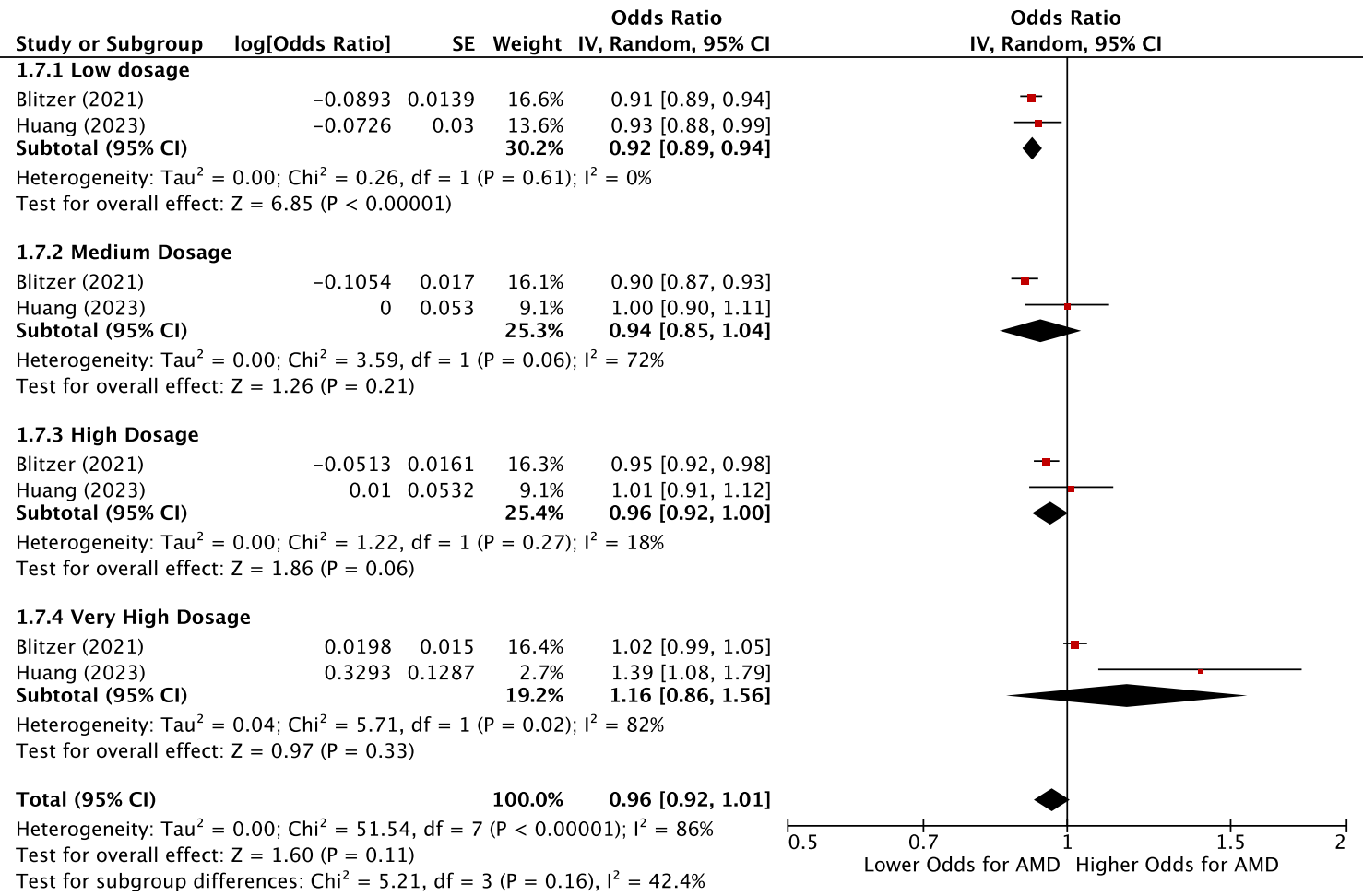

Supplement: Figure S4 [file mmc4.pdf]

Supplementary Figure 5: Odds Ratio Forest Plot, Sensitivity Analysis

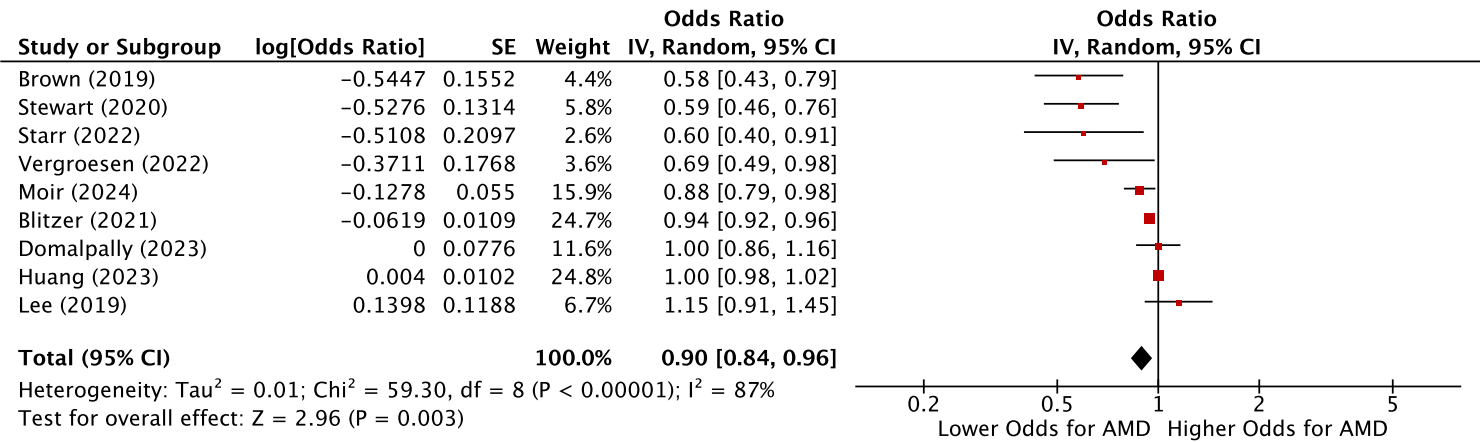

Supplement: Figure S5 [file mmc5.pdf]
