# Supplementary material for: Potential Efficacy of Metformin for Age-Related Macular Degeneration: A Systematic Review and Meta-Analysis
Source: Ophthalmol Sci. 2025 Feb 15;5(4):100741. doi: 10.1016/j.xops.2025.100741 (PMC11994399; doi:10.1016/j.xops.2025.100741)
Supplement: Table S1 [file mmc6.pdf]

Supplementary Table 1: Search Strategies in Ovid MEDLINE (a), Ovid Embase (b), Cochrane Library (c), and Web of Science (d)

a. Search strategy in MEDLINE (via Ovid MEDLINE(R), 1946 to present; search date: 5/02/2024)

#	Search syntax	Citations found
1	((macul* or retina* or choroid*) adj4 degenera*).tw.	46524
2	((macul* or retina* or choroid*) adj4 neovascular*).tw.	20895
3	((macul* or geograph*) adj4 atroph*).tw.	6541
4	maculopath*.tw.	5866
5	(AMD or ARMD or CNV or GA).tw.	115005
6	exp Macular Degeneration/	31274
7	exp Retinal Degeneration/	52662
8	exp Choroidal Neovascularization/	6977
9	exp Geographic Atrophy/	1147
10	exp Retinal Neovascularization/	3452
11	1 or 2 or 3 or 4 or 5 or 6 or 7 or 8 or 9 or 10	167491
12	metformin*.tw.	29652
13	exp Metformin/	18645
14	11 or 12	31556
15	13 and 14	166

b. Search strategy in Embase (via Ovid Embase, 1974 to present; search date: 5/02/2024)

#	Search syntax	Citations found
1	((macul* or retina* or choroid*) adj4 degenera*).tw.	75774
2	((macul* or retina* or choroid*) adj4 neovascular*).tw.	25552
3	(geograph* adj4 atroph*).tw.	3839
4	exp retina degeneration/	78625
5	exp retina neovascularization/	7509
6	exp retina maculopathy/	88833
7	exp macular degeneration/	47132
8	exp retina macula age related degeneration/	28876
9	exp retina macula degeneration/	47132
10	exp geographic atrophy/	2904
11	maculopath*.tw.	12216
12	1 or 2 or 3 or 4 or 5 or 6 or 7 or 8 or 9 or 10 or 11	142720
13	metformin*.tw.	95859
14	exp metformin/	91519
15	12 or 13	95859
16	14 and 15	552

c. Search strategy in Cochrane Library (via Cochrane Collaboration; search date: 5/02/2024)

#	Search syntax	Citations found
1	MeSH descriptor: [Macular Degeneration] explode all trees	3626
2	MeSH descriptor: [Geographic Atrophy] explode all trees	208
3	MeSH descriptor: [Retinal Degeneration] explode all trees	3815
4	MeSH descriptor: [Retinal Neovascularization] explode all trees	113
5	MeSH descriptor: [Choroidal Neovascularization] explode all trees	579
6	(macula* or retina* or choroid*) near/4 degenerat*	4342
7	(macula* or retina* or choroid*) near/4 neovascul*	2729
8	geograph* near/4 atroph*	551
9	maculopath*	527
10	AMD or ARMD or CNV or GA	26333
11	#1 or #2 or #3 or #4 or #5 or #6 or #7 or #8 or #9 or #10	30232
12	Metformin*	14374
13	MeSH descriptor: [Metformin] explode all trees	5524
14	#12 or #13	14374
15	#11 and #14	145

d. Search strategy in Web of Science Core Collection (2015 to present; search date: 5/02/2024)

#	Search syntax	Citations found
1	(((((TS=((macula* or retina* or choroid*) near/4 degenerat*)) OR TS=((macula* or retina* or choroid*) near/4 neovascul*)) OR TS=(maculopath*)) OR TS=(geograph* near/4 atroph*)) OR TS=(AMD or ARMD or CNV or GA)) AND TS=(metformin*)	209
