# Supplementary material for: Potential Efficacy of Metformin for Age-Related Macular Degeneration: A Systematic Review and Meta-Analysis
Source: Ophthalmol Sci. 2025 Feb 15;5(4):100741. doi: 10.1016/j.xops.2025.100741 (PMC11994399; doi:10.1016/j.xops.2025.100741)
Supplement: Table S2 [file mmc7.pdf]

Supplementary Table 2: Covariate Data

Source (Country)	Age	Sex	BMI	Socioeconomic Data	Smoking	Anemia	DM	DR	HTN	Hyperlipidemia	CCI	Other Variables
Aggarwal et al, 2024 (US)	•	•			•	•			•	•	•	Statins, Antidiabetics, US Region
Blitzer et al, 2021 (US)	•	•			•	•	•	•	•	•	•	Statins, Antidiabetics, US Region
Brown et al, 2019 (US)	•	•	•	•		•	•		•		•	Ocular Comorbities, Statins, SSRI, Antidepressants, Antidiabetics, Antihypertensives
Chen et al, 2019 (Taiwan)	•	•	•					•	•	•		CKD, Obesity, Antidiabetics, Antihypertensives, Race/Ethnicity, CAD
Domalpally et al, 2023 (US)	•	•	•	•	•		•	•				HbA1c, Dietary Protein, Fasting Glucose, Statin, Antidiabetics, Race/Ethnicity
Eton et al, 2022 (US)	•	•		•		•			•			NPDR, CKD, Hypercholesterolemia, ESRD, DCSI, HbA1c, Statins, Race, US Region
Gokhale et al, 2023 (UK)	•	•	•	•	•				•		•	HbA1c, CKD, Hypothyroidism, CVD, Statins, Antidiabetics, Ethnicity
Huang et al, 2023 (Taiwan)	•	•		•			•			•		Cerebrovascular Disease, Obesity, Hyperuricemia, Alcoholism, NAFLD, RA, Hypothyroidism, HBV, HCV, Sleep Disturbance, CKD, SLE, Migraines
Jiang et al, 2022 (China)	•	•	•		•		•	•	•	•		HbA1c, Fasting Blood Glucose, Total Cholesterol, Blood Uric Acid, Blood Creatinine
Kaufmann et al, 2023 (US)	•	•			•			•	•	•	•	Statins, Antidiabetics
Khanna et al, 2024 (US)	•	•			•		•	•	•	•	•	Obesity, Statins, Antidiabetics
Lee et al, 2019 (Korea)	•	•		•			•		•	•	•	Cerebrovascular Disease, Liver Desease, Myocardial Infarction, Peripheral Vascular Diseases, Alpha-Blokers, Statins, Ace Inhibitors, ARBs
Shaw et al, 2024 (US)	•	•			•	•		•	•	•	•	Region
Starr et al, 2022 (US)	•	•	•		•		•		•	•		Caucasian, Congestive Heart Failure, Antidiaabetics, CAD
Stewart et al, 2020 (US)	•	•		•	•							Race/Ethnicity
Tseng et al, 2023 (Taiwan)	•	•		•	•				•			Dyslipidemia, Taiwan Region, Obesity, Diabetic Complications, Antidiabetic Drugs, Statins, Various Chronic Obstructive Pulmonary Disease
Vergroesen et al, 2022 (US)	•	•	•		•		•					Statins, Antihypertensives

BMI, body mass index; DM, diabetes mellitus; DR, diabetic retinopathy; HTN, hypertension; CCI, Charlson Comorbidity Index; CKD, chronic kidney disease; CAD, coronary artery disease; CVD, cardiovascular disease; NAFLD, non-alcoholic fattly liver disease; RA, rheumatoid arthritis, HBV, hepatitis B virus; HCV, hepatitus C virus
